# Oil-Contaminated Soil Remediation with Biodegradation by Autochthonous Microorganisms and Phytoremediation by Maize (*Zea mays*)

**DOI:** 10.3390/molecules28166104

**Published:** 2023-08-17

**Authors:** Katarzyna Wojtowicz, Teresa Steliga, Piotr Kapusta, Joanna Brzeszcz

**Affiliations:** Oil and Gas Institute—National Research Institute, ul. Lubicz 25 A, 31-503 Krakow, Poland; teresa.steliga@inig.pl (T.S.); piotr.kapusta@inig.pl (P.K.); joanna.brzeszcz@inig.pl (J.B.)

**Keywords:** biodegradation, biopreparation, ecotoxicology, microorganisms, petroleum contaminants, phytoremediation, γ-PGA-polyglutamic acid

## Abstract

Biological methods are currently the most commonly used methods for removing hazardous substances from land. This research work focuses on the remediation of oil-contaminated land. The biodegradation of aliphatic hydrocarbons and PAHs as a result of inoculation with biopreparations B1 and B2 was investigated. Biopreparation B1 was developed on the basis of autochthonous bacteria, consisting of strains *Dietzia* sp. IN118, *Gordonia* sp. IN101, *Mycolicibacterium frederiksbergense* IN53, *Rhodococcus erythropolis* IN119, *Rhodococcus globerulus* IN113 and *Raoultella* sp. IN109, whereas biopreparation B2 was enriched with fungi, such as *Aspergillus sydowii*, *Aspergillus versicolor*, *Candida* sp., *Cladosporium halotolerans*, *Penicillium chrysogenum*. As a result of biodegradation tests conducted under ex situ conditions for soil inoculated with biopreparation B1, the concentrations of TPH and PAH were reduced by 31.85% and 27.41%, respectively. Soil inoculation with biopreparation B2 turned out to be more effective, as a result of which the concentration of TPH was reduced by 41.67% and PAH by 34.73%. Another issue was the phytoremediation of the pre-treated G6-3B2 soil with the use of *Zea mays*. The tests were carried out in three systems (system 1—soil G6-3B2 + *Zea mays*; system 2—soil G6-3B2 + biopreparation B2 + *Zea mays*; system 3—soil G6-3B2 + biopreparation B2 with γ-PGA + *Zea mays*) for 6 months. The highest degree of TPH and PAH reduction was obtained in system 3, amounting to 65.35% and 60.80%, respectively. The lowest phytoremediation efficiency was recorded in the non-inoculated system 1, where the concentration of TPH was reduced by 22.80% and PAH by 18.48%. Toxicological tests carried out using Phytotoxkit^TM^, Ostracodtoxkit^TM^ and Microtox^®^ Solid Phase tests confirmed the effectiveness of remediation procedures and showed a correlation between the concentration of petroleum hydrocarbons in the soil and its toxicity. The results obtained during the research indicate the great potential of bioremediation practices with the use of microbial biopreparations and *Zea mays* in the treatment of soils contaminated with petroleum hydrocarbons.

## 1. Introduction

Human activity has been altering the environment for ages, reducing its resources, changing its natural properties and introducing foreign substances not present in the natural conditions. Oil and gas mining uses up valuable minerals, involving large amounts of various materials and energy and producing a significant amount of waste and causing irreversible changes in the natural environment. Petroleum products are one of the main sources of soil pollution appearing due to oil mining work. Petroleum waste has an adverse effect on plant production and is dangerous to people and animals. The introduction of petroleum waste into the ecosystems disturbs the natural cycles of matter and energy, reducing the water storage capacity of the soil and hindering air exchange between the soil and the atmosphere. Additionally, the large number of different specific properties of petroleum pollutants and the products of their biological and physico-chemical transformations in the natural environment make it very difficult to identify the environmental consequences of environmental pollution and select the correct remediation technology and the methods for determining and monitoring the progress of the remediation of contaminated soil [1]. At the moment, the most popular methods for the remediation of petroleum-contaminated soil include biological methods, which have a wide range of applications and are highly efficient and fairly inexpensive. However, biological methods have their limitations, including the fact that high concentrations of pollutants in the soil may exceed the levels tolerated by the organisms used in the process. For this reason, in some cases, it is necessary to conduct staged remediation of soil using multiple remediation methods.

Biodegradation using biopreparations made with microorganisms isolated from areas naturally occurring in the remediated region is one of the most effective and frequently used methods for remediating petroleum-contaminated soil. It is estimated that out of the native microbiological population, approx. 10^5^ to 10^6^ have properties, which enable the biodegradation of petroleum hydrocarbons [2]. However, it should be noted that most bacteria, fungi and yeasts metabolise petroleum products to a limited extent. *Desulfosarcina* sp. [3], *Mycobacterium* sp., *Pseudomonas butanovora* [4], *Pseudonocardia* sp. [5] bacteria are effective at breaking down C_2_–C_8_ alkanes, whereas *Acinetobacter baumannii* [6], *Alcanivorax borkumensis* [7], *Collimonas* sp., *Dietzia* sp. [8], *Flavobacterium* sp., *Pseudomonas* sp. [9], *Geobacillus thermodenitrificant* [10], *Gordonia sihwensis* [11], *Mycobacterium* sp. [12], *Mycobacterium frederiksbergense* IN53, *Acinetobacter* sp. IN47 [13,14], *Nocardioides albus, Ochrobactrum* sp., *Oleispira antarctica* [15], *Pseudomonas aeruginosa* [16,17,18,19,20], *Pseudomonas* spp. *Alphaproteobacteria* and *Betaproteobacteria* [21], *Pseudomonas citronellolis* [22], *Pseudomonas stutzeri* [15,23], *Rhodococcus* sp. [24], *Rhodococcus coprophilus* [25], *Rhodococcus ruber* [26] or *Stenotrophomonas maltophilia* are used in biodegradation tests of alkanes with a carbon chain length of C_10_–C_36_, oils and asphaltenes. In addition, it was confirmed that the fungal strains *Aspergillus fumigatus*, *Aspergillus, terreus*, *Fusarium solani*, *Gliomastix* sp., *Paecilomyces* sp. and *Verticillum* sp. have the ability to degrade short-chain n-alkanes [27,28]. Hydrocarbons with a carbon chain length of C_10_–C_36_ are also broken down by fungi *Penicillium chrysogenum* [29], *Penicillium Citrinum* [27], *Candida* sp. [30], *Candida lipolytica* [28], *Candida parapsilosis, Candida krusei, Candida famata* and *Rhodotorula* spp. [31], *Aspergillus flavus, Fusarium* sp., *Scopulariopsis* sp. [27].

The most popular microorganisms capable of breaking down polycyclic aromatic hydrocarbons include the *Achromobacter xylosoxidans* [32], *Aeribacillus pallidus* [33], *Alcaligenes faecalis*, *Agmenellum quadruplicatum* [34], *Bacillus Licheniformis*, *Bacillus Mojavensis* [35], *Bacillus thuringiensis*, *Brevundimonas* sp. [36], *Burkholderia* sp., *Cycloclasticus* sp. [37], *Gordona sputi* [38,39], *Micrococcus* sp. [40], *Mycobacterium* sp. [41,42], *Mycobacterium vanbaalenii* [43,44,45], *Neptunomonas naphthovoran* [46], *Nocardioides* sp., *Nocardia*, *Oscillatoria* [47], *Pleurotus cornucopiae* [48], *Pseudomonas* sp. [49,50], *Pseudomonas aeruginosa* [50], *Pseudomonas putida* [51], *Pseudomonas stutzeri* [52], *Rhodococcus* sp. [53], *Rhodococcus erythropolis* [54,55,56], *Sphingobium* and *Novosphingobium*, *Sphingomonas* sp. [57,58], *Sphingomonas yanoikuyae* [59], *Staphylococcus warneri* and *Bacillus pumilus* [60], *Stenotrophomonas* sp. [61], *Stenotrophomonas maltophilia* [62], *Streptomyces* sp. [63] bacteria and fungi *Aspergillus versicolor* [64], *Aspergillus sydowii* [65], *Candida* sp. [30], *Clodosporium* sp. [66], *Penicillium* sp., *Fusarium solani* [67,68]. For this reason, it is preferred to use biopreparations containing several to several dozen strains of bacteria and fungi to ensure the broadest possible spectrum of action. The microorganisms contained in the biopreparations should be specially selected from the natural environment, which significantly improves the efficiency of pollutant removal [69,70] and helps avoid antagonistic reactions of the native micro-flora of the soil to the foreign microbial cultures. It is also necessary to bear in mind that the microbes introduced into the soil cannot be pathogenic to plants and animals, and they should not produce toxic substances, should not be resistant to antibiotics and should not be involved in the frequent exchange of genes, which encode undesirable properties [71,72,73,74]. At the moment, there are more and more studies combining several methods of soil bioremediation to ensure the highest possible efficiency. Interesting solutions, which have been successfully applied, include the combination of biodegradation of petroleum products by inoculation with a biopreparation with phytoremediation.

Biological methods using plants (phytoremediation) mainly rely on the tolerance for high concentrations of toxic substances, absorption, accumulation and metabolism of hazardous substances in large quantities (in the plant’s own organs) and stabilisation of pollutants in the soil or transformation of toxic substances in the environment into non-toxic compounds [75]. Organic pollutants are absorbed by the plants together with water by simple diffusion. The diffusion of petroleum products into the plant, however, is hindered if most ingredients of crude oil are hydrophobic compounds, which are highly lipophilic and have large molecular masses. For this reason, the absorption of lipophilic substances by plants is positively correlated with the lipid content of the root epidermis. However, plants may release surface-active agents, such as low-molecular-weight organic acids or polypeptides, which reduce surface tension and emulsify hydrophobic substances, increasing bioavailability [76,77,78,79,80,81,82,83]. The plants used for phytoremediation should be tolerant of high concentrations of xenobiotics, a high degree of accumulation or biodegradation of pollutants, even with a fairly low pollution level, the ability to accumulate multiple pollutants at the same time, quick growth, high biomass production and resistance to diseases and pests and difficult environmental conditions [75,84]. The most often cited species of haptophytes include the tall sedge (*Carex hirta*), Kentucky bluegrass (*Poa pratensis*), perennial ryegrass (*Lolium perenne*), broadleaf plantain (*Plantago major*) and common nettle (*Urtica dioica*), which tolerate petroleum pollutants in the soil in the range of 204–504 g/kg, 112–320 g/kg, 132–166 g/kg, up to 470 g/kg and up to 506 g/kg, respectively [85]. Other haptophytes include the common toadflax (*Linaria vulgaris*) [86], European dewberry (*Rubus caesius*), tufted grass (*Holcus lanatus*) [87], white clover (*Trifolium repens*) [88], sweet yellow clover (*Melilotus officinalis*) [89] and tall fescue (*Festuca arundinacea*) [90]. Further plant species, which can successfully be used in the phytoremediation of petroleum compounds, include the marvel of Peru (*Mirabilis jalapa*) [91], alfalfa (*Medicago sativa*) [92], perennial ryegrass (*Lolium perenne, Lolium multiflorum*) [93,94], common bird’s-foot trefoil (*Lotus corniculatus*) [94,95], sorghum (*Sorghum bicolor*) [96], maize (*Zea mays*) [97,98,99], Bermuda grass (*Cynodon dactylon*) [100], nodding beggarticks (*Bidens cernua*) [101], common blanketflower (*Gaillardia aristata*) [102], eastern purple coneflower (*Echinacea purpurea*) [102,103] and grasses and leguminous plants [104,105,106]. Since the plants used for phytoremediation should grow fast, have a deep and extensive root system and fairly high biomass, trees such as poplars (*Populus* L.) and birches (*Salix* L.) [107,108] are increasingly often considered as plants, which can be used for the bioremediation of petroleum-contaminated soil. Additionally, the effectiveness of phytoremediation largely depends on the soil conditions, which affect the growth of plant organisms. For this reason, recent years saw increased research on the ways of improving the efficiency of phytoremediation by combining it with biodegradation using biopreparations made with native microorganisms and biopreparation additives. One of the compounds, which can increase the efficiency of phytoremediation of petroleum-contaminated soil is γ-PGA.

γ-PGA, i.e., polyglutamic acid, is a chemical compound produced by bacteria of the *Bacillus* genus, including in particular *Bacillus amyloliquefaciens, Bacillus licheniformis, Bacillus megaterium, Bacillus subtilis* and *Bacillus anthracis* [109]. γ-PGA is highly soluble in water, non-toxic to living organisms and readily biodegradable, and it can be used as a natural additive to fertilisers [110,111,112,113]. Additionally, γ-PGA is a readily available source of carbon, which significantly affects the growth of plant organisms. The satisfactory effects of the addition of γ-PGA to biopreparations made with native bacteria on the efficiency of biodegradation of petroleum hydrocarbons in the soil in areas of drilling waste pits [55,114]; the impact of γ-PGA on the removal of oil from marine sediments [115] and trichloroethane from the aquifer [116]; and the use of γ-PGA for the remediation of soil contaminated with heavy metals [117,118,119] were the reasons for the decision to experimentally test the possibilities of using this compound in the phytoremediation process.

## 2. Results

### 2.1. Biodegradation of Hydrocarbons Using Biopreparations

Biodegradation tests of petroleum pollutants using the ex situ prism method demonstrated that the addition of fungi to the biopreparation increased the efficiency of biodegradation of both aliphatic hydrocarbons and aromatic hydrocarbons. After three months of tests, the TPH content was reduced from 4527.70 mg/kg ds to 3090.60 mg/kg ds (in soil inoculated with biopreparation B1) and to 2640.83 mg/kg ds (in soil inoculated with biopreparation B2).

**Figure 1:**
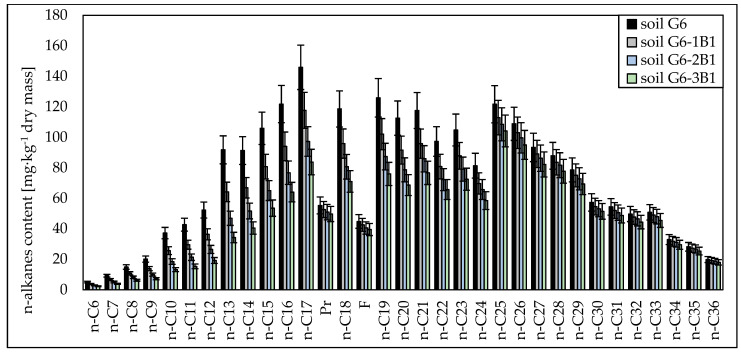
Concentrations of n-alkanes in soil inoculated with biopreparation B1 over 3 months of observation (repetition number *n* = 7–10, *p* < 0.05).

**Figure 2:**
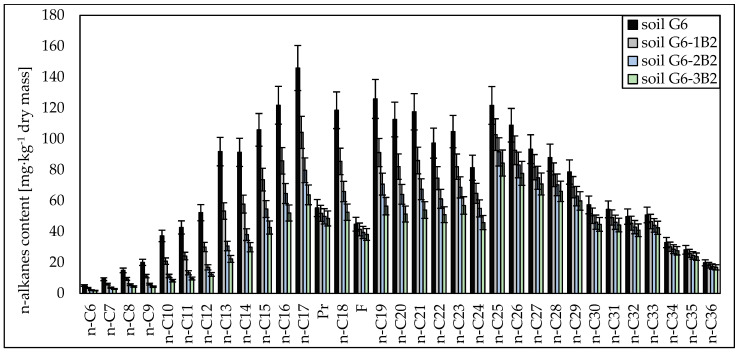
Concentrations of n-alkanes in soil inoculated with biopreparation B2 over 3 months of observation (repetition number *n* = 7–10, *p* < 0.05).

Chromatographic analyses showed that both in the case of soil inoculated with the biopreparation made with bacteria and the biopreparation made with bacteria, yeasts and fungi, aliphatic hydrocarbons with a carbon chain length of n-C_10_–n-C_21_ were the most readily biodegradable. Their degrees of biodegradation in soil inoculated with biopreparation B1 ranged from 18.49% to 31.00% (after 1 month), from 26.93% to 50.37% (after 2 months) and from 34.78% to 64.58% (after 3 months), and in soil inoculated with biopreparation B2, the degrees of biodegradation of n-C_10_–n-C_21_ hydrocarbons were 26.83% to 43.86% (after 1 month), 42.63% to 69.69% (after 2 months) and 54.08% to 78.00% (after 3 months), respectively. A slightly smaller biodegradation efficiency was recorded for light hydrocarbons with a carbon chain length of n-C_6_–n-C_9_, which ranged from 53.96% to 63.99% in G6-3B1 soil and from 78.50% to 65.17% in G6-3B2 soil after the tests. Significant differences in the biodegradation of aliphatic hydrocarbons in soil inoculated with biopreparations B1 and B2 were recorded for hydrocarbons with a carbon chain length of n-C_22_–n-C_30_ and n-C_31_–n-C_36_. Their biodegradation in soil inoculated with the biopreparation made with bacteria was much slower than in soil inoculated with the biopreparation made with bacteria, yeasts and fungi. The degree of biodegradation of hydrocarbons with a carbon chain length of n-C_22_–n-C_30_ ranged from 4.76% to 16.93% (soil G6-1B1), 7.24% to 25.76% (soil G6-2B1), 10.25% to 32.44% (soil G6-3B1), 12.28% to 23.34% (soil G6-1B2), 19.52% to 37.08% (soil G6-2B2) and 22.05% to 47.63% (soil G6-3B2). Heavy hydrocarbons containing more than 30 carbon atoms per molecule were broken down the most. Inoculation with biopreparation B1 decreased their content in the soil by 2.87% to 4.41% (after 1 month), 5.54% to 7.14% (after 2 months) and 9.23% to 10.67% (after 3 months). The biodegradation of heavy hydrocarbons was much more efficient in soil inoculated with biopreparation B2. The content of hydrocarbons in the n-C_31_–n-C_36_ range was reduced by 7.79% to 9.65% after 1 month, by 12.38% to 15.33% after 2 months and by 14.77% to 18.65% after 3 months. The content of unidentified hydrocarbons after 3 months of biodegradation tests using the prism method decreased by 31.44% (soil G6-3B1) and 37.97% (soil G6-3B2). The specific progress of the biodegradation of aliphatic hydrocarbons in soil G6 inoculated with biopreparations B1 and B2 is shown in Figure 1 and Figure 2.

**Figure 3:**
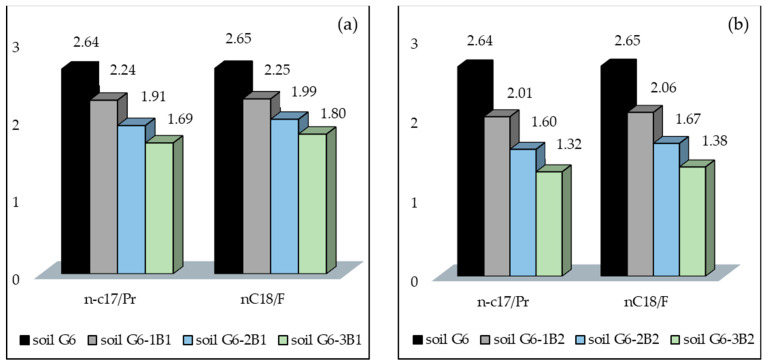
(**a**) n-C17/Pr and (**b**) n-C18/F ratios in soil inoculated with biopreparations B1 and B2 over subsequent months of remediation using the ex situ method (repetition number *n* = 7–10, *p* < 0.05).

Another important parameter, which can be used to determine the efficiency of biodegradation of petroleum pollutants in soil, is the change in n-C_17_/Pr and n-C_18_/F ratios. After the test, the n-C_17_/Pr ratio was lower in soil inoculated with biopreparation B2 than in soil inoculated with biopreparation B1 by 0.29. Additionally, the n-C_17_/Pr ratio in G6-3B2 soil was lower by 0.30 in comparison with G6-3B1 soil. The n-C_17_/Pr and n-C_18_/F biodegradation ratios are indicative of a higher efficiency of biodegradation of petroleum hydrocarbons in soil inoculated with biopreparation B2. The values of n-C_17_/Pr and n-C_18_/F biodegradation ratios are shown in Figure 3.

**Figure 4:**
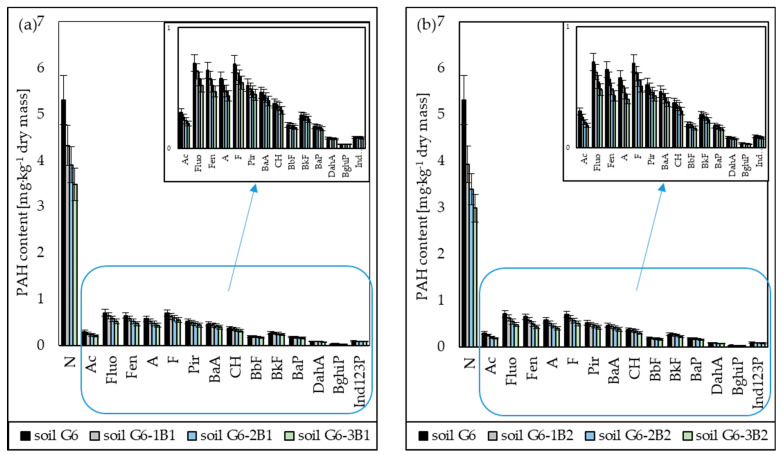
List of PAH concentrations in soil inoculated with biopreparations (**a**) B1 and (**b**) B2 over 3 months (ex situ method) (repetition number *n* = 7–10, *p* < 0.05). N—naphthalene, Ac—acenaphthene, Fluo—fluorene, Phen—phenanthrene, A—anthracene, F—Fluoranthene, Pir—pyrene, BaA—benzo(a)anthracene, CH—chrysene, BbF—benzo(b)fluoranthene, BkF—benzo(k)fluoranthene, BaP—benzo(a)pyrene, DahA—dibenzo(a,h)anthracene, BghiP—benzo(g,h,i)perylene, Ind123P—indeno(1,2,3-c,d)pyrene.

The PAH biodegradation tests in soil inoculated with biopreparations B1 and B2 showed that the presence of fungi in the biopreparation increased the efficiency of PAH biodegradation. This is confirmed by the results of the chromatographic analyses, which showed that the concentration of naphthalene in soil inoculated with biopreparation B1 in subsequent months of the tests decreased by 18.54% (after 1 month), 26.54% (after 2 months) and 34.49% (after 3 months), and in soil inoculated with biopreparation B2 by 25.96% (after 1 month), 36.11% (after 2 months) and 43.81% (after 3 months). Thus, the difference in the degrees of biodegradation of naphthalene in soil inoculated with biopreparations B1 and B2 after the tests amounted to 9.32%, which is a significant level, considering the short duration of the tests. Additionally, the chromatographic analyses showed that the degree of biodegradation decreased along with the increase in the number of rings in the PAH molecule. The 3-ring PAHs (acenaphthene, fluorene, phenanthrene, anthracene) turned out to be slightly less biodegradable than naphthalene, and their degrees of biodegradation ranged from 10.02% to 13.86% (soil G6-1B1), from 17.73% to 23.44% (soil G6-2B1), from 24.40% to 30.82% (soil G6-3B1), from 12.08% to 16.70% (soil G6-1B2), from 22.44% to 29.67% (soil G6-2B2) and from 30.12% to 38.05% (soil G6-3B2). The degrees of reduction in 4-ring PAHs (fluoranthene, pyrene, benzo[a]anthracene, chrysene) were much lower, and after the tests, they ranged from 15.22% to 21.71% for the soil inoculated with biopreparation B1 and from 18.79% to 26.80% for the soil inoculated with biopreparation B2. The 5-ring and 6-ring PAHs were the least biodegradable. The degrees of biodegradation of 5-ring PAHs in soil inoculated with biopreparations B1 and B2 after 3 months of tests ranged from 11.42% to 11.46% and from 16.56% to 16.61%, respectively, and the degrees for 6-ring PAHs ranged from 7.96% to 8.37% (soil G6-3B1) and from 14.77% to 15.27% (soil G6-3B2), respectively. The specific progress of biodegradation of PAHs in soil G6 inoculated with biopreparations B1 and B2 is shown in Figure 4.

The content of the individual groups of hydrocarbons before and after the biodegradation tests of petroleum products by inoculation with biopreparations made with native microorganisms (biopreparations B1 and B2) is shown in Supplementary Materials (Table S1).

### 2.2. Phytoremediation

**Figure 5:**
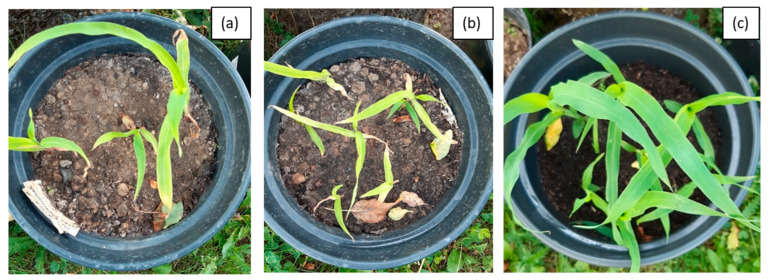
Growth and degree of germination of Zea mays after 1 month of research on phytoremediation of soil G6-3B2 under the tested systems: (**a**) system 1, (**b**) system 2, (**c**) system 3.

**Figure 6:**
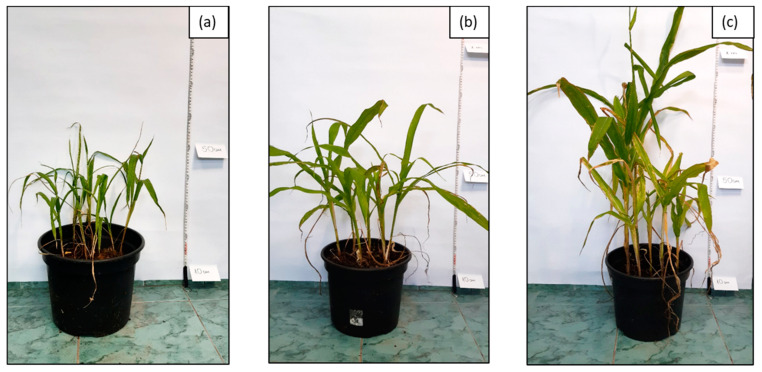
Appearance of *Zea mays* after 6 months of research on phytoremediation of soil G6-3B2 under the studied systems: (**a**) system 1, (**b**) system 2, (**c**) system 3.

**Figure 7:**
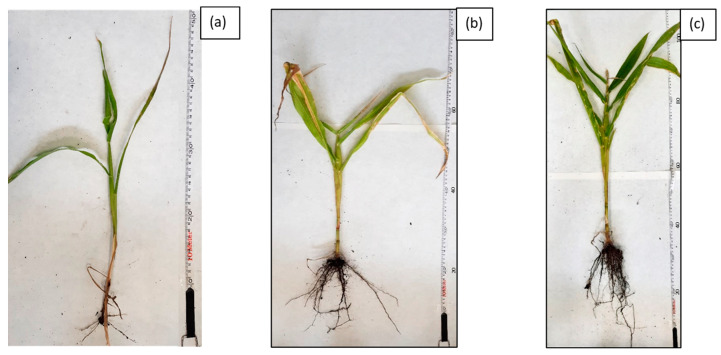
Morphology of selected *Zea mays* individuals after completion of G6-3B2 soil phytoremediation studies under the studied systems: (**a**) system 1, (**b**) system 2, (**c**) system 3.

The first stage of phytoremediation tests was the morphological analysis of plant organisms in the tested systems. Significant differences were observed in the development of the individual specimens of maize in soil not inoculated with biopreparations, soil inoculated with biopreparation B2 and soil inoculated with biopreparation B2 with added γ-PGA. An analysis of the morphological properties of maize in the first and sixth month of phytoremediation tests of petroleum-contaminated soil shows that the plants grew best in the system inoculated with biopreparation B2 with added γ-PGA. This is indicated by the best growth of the plants, the colour of the leaves and the development of the root. Additionally, all the maize seeds sown germinated in the first month of the tests. After 6 months of phytoremediation in system 3, the individual maize specimens reached sizes from 90 to 120 cm, and their root system was well developed. Additionally, the size of the individual leaves ranged from 30 to 50 cm, and they had an intensely green colour. Additionally, maize sown in soil inoculated with biopreparation B2 with added γ-PGA was able to develop more leaves than in soil inoculated with the biopreparation alone and in soil, which was not inoculated. In system 2, six out of eight seeds germinated, but the growth of the individual specimens was much slower. Additionally, in comparison with the system inoculated with biopreparation B2 with added γ-PGA, the individual maize specimens were less developed (smaller leaves, poorly developed root system). The maize in soil inoculated with biopreparation B2 after 6 months of bioremediation tests reached a height of 50 to 80 cm and had a moderately developed root system. The leaves of the plant reached a length of 20 to 30 cm and had a light green colour. The development of maize was the slowest by a wide margin in system 1. Out of eight seeds, only three germinated, and their sizes were much smaller than the sizes of specimens in systems 2 and 3. The sizes of the individual plants at the end of phytoremediation in system 1 ranged from 30 to 50 cm, and their root systems were very poorly developed, which hindered the efficient remediation of petroleum-contaminated soil. The appearance of the selected maize specimens in soil G6-3B2(F1), G6-3B2(F2) and G6-3B2(F3) is shown in Figure 5, Figure 6 and Figure 7.

The morphological analysis only enables an indicative determination of the effectiveness of phytoremediation. The observations of the development of maize suggest that inoculation with biopreparation B2 increases the efficiency of phytoremediation of petroleum-contaminated soil, and the use of the γ-PGA additive with biopreparation B2 increases it further. This hypothesis is confirmed by the results of chromatographic analyses, which enable a specific determination of the changes in the concentration of hydrocarbons under the tested systems.

The developed chromatographic methods for the determination of aliphatic hydrocarbons in contaminated soil enable the identification of n-alkanes in contaminated soil after phytoremediation. As a result of the tests conducted, the highest degree of biodegradation of TPH and PAHs was achieved in the system inoculated with biopreparation B2 with added γ-PGA, and the least efficient biodegradation was recorded in the system not inoculated with biopreparations. This indicates that phytoremediation alone only slightly increases the efficiency of remediation of petroleum-contaminated soil, whereas the combination of inoculation with biopreparation with phytoremediation significantly improves its efficiency.

**Figure 8:**
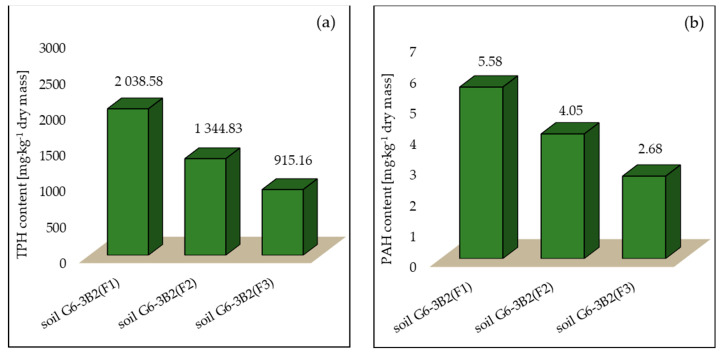
Concentration of (**a**) TPH and (**b**) PAHs in soils after completion of the phytoremediation process using *Zea mays* (repetition number *n* = 7–10, *p* < 0.05).

As a result of phytoremediation using maize, the concentration of TPH in the tested systems after 6 months of tests decreased by 22.81% (system 1), 49.08% (system 2) and 65.35% (system 3), whereas the efficiency of PAH phytoremediation was 18.48% in soil G6-3B2(F1), 40.74% in soil G6-3B2(F2) and 60.80% in soil G6-3B2(F3). The TPH and PAH concentrations in the tested systems at the end of phytoremediation are shown in Figure 8.

**Figure 9:**
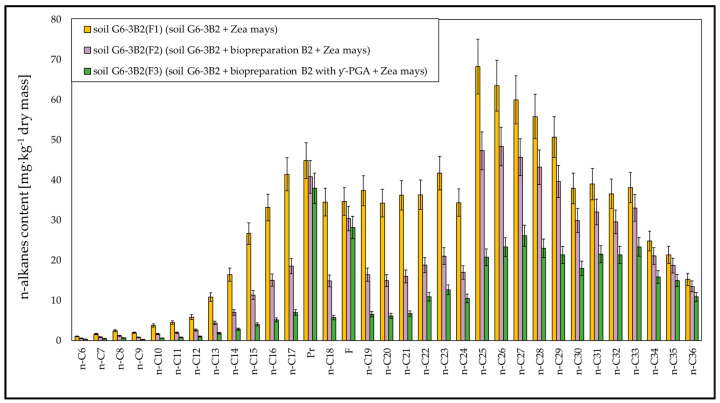
N-alkanes content in the tested soils after completion of the phytoremediation process. Soil G6-3B2 was the control (repetition number *n* = 7–10, *p* < 0.05).

The results of chromatographic analyses showed that during phytoremediation, the highest efficiency was recorded for the biodegradation of aliphatic hydrocarbons with a carbon chain length of n-C_10_–n-C_21_, for which the degrees of biodegradation ranged from 32.99% to 53.93% (soil G6-3B2(F1)), from 70.39% to 80.21% (soil G6-3B2(F2)) and from 87.51% to 92.36% (soil G6-3B2(F3)). A satisfactory degree of biodegradation was also observed for light hydrocarbons with a carbon chain length of n-C_6_–n-C_9_ and hydrocarbons in the n-C_22_–n-C_30_ range. In both cases, phytoremediation using maize was most efficient in system 3, i.e., soil inoculated with a mixture of biopreparation B2 and γ-PGA. The degrees of biodegradation of n-C_6_–n-C_9_ ranged from 80.00% to 92.97% and for n-C_22_–n-C_30_—from 59.67% to 78.54%. Hydrocarbons with fewer than 30 carbon atoms per molecule were broken down to a smaller extent, and their degrees of biodegradation for systems 1, 2 and 3 ranged from 9.58% to 11.86%, 21.36% to 27.54% and 35.52% to 51.34%, respectively. The content of unidentified hydrocarbons after 6 months of phytoremediation decreased by 21.69% in soil G6-3B2(F1), by 48.47% in soil G6-3B2(F2) and by 60.67% in soil G6-3B2(F3). A comparison of changes in the content of aliphatic hydrocarbons in soil G6-3 after phytoremediation using maize is shown in Figure 9.

**Figure 10:**
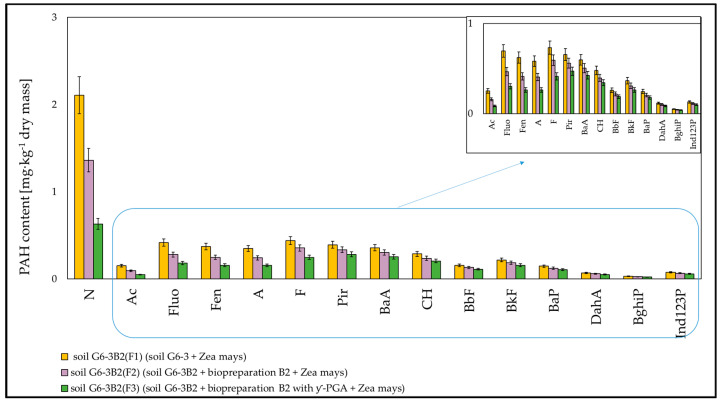
PAHs content in the tested soils after completion of the phytoremediation process. Soil G6-3B2 was the control (repetition number *n* = 7–10, *p* < 0.05).

The efficiency of the removal of polycyclic aromatic hydrocarbons from the soil by phytoremediation using maize was the highest in the system inoculated with biopreparation B2 with added γ-PGA, slightly lower in the system inoculated with biopreparation alone and significantly lower in the system, which was not inoculated. Out of the determined polycyclic aromatic hydrocarbons, the highest degree of biodegradation after 6 months of the experiment was recorded for naphthalene, and it amounted to 29.39% in soil G6-3B2(F1), 54.34% in soil G6-3B2(F2) and 78.87% in soil G6-3B2(F3). Slightly worse biodegradation was recorded for the 3-ring PAHs (Ac, Fluo, Fen, A), whose degrees of biodegradation ranged from 12.99% to 18.91% in system 1, from 41.70% to 49.10% in system 2 and from 60.99% to 72.84% in system 3. Through biodegradation combined with phytoremediation, the concentrations of 4-ring PAHs (F, Pir, BaA, CH) were reduced in systems 1, 2 and 3 by 5.21% to 13.98%, 20.02% to 30.55% and 32.20% to 51.50%, respectively. The 5-ring and 6-ring PAHs were less readily biodegradable, and their degrees of biodegradation ranged from 3.39% to 4.67% in soil G6-3(1), from 17.97% to 19.92% in soil G6-3(2) and from 26.67% to 31.09% in soil G6-3(3). The specific progress of biodegradation of PAHs in soil G6-3 subjected to phytoremediation under the tested systems is shown in Figure 10.

The content of the tested groups of petroleum hydrocarbons in soil at the end of the phytoremediation process (6 months) under three tested systems is shown in Supplementary Materials (Table S2).

### 2.3. Toxicological Analyses

Toxicological tests were conducted on soil samples before the remediation process (initial soil—G6), at the end of biodegradation using biopreparations B1 (soil G6-3B1) and B2 (soil G6-3B2) (3 months) and at the end of phytoremediation under the tested process systems (phytoremediation, inoculation with biopreparation B2 and inoculation with a mixture of biopreparation B2 and γ-PGA solution) (soil G6-3B2(F1), G6-3B2(F2), G6-3B2(F3)). The results enabled the determination of changes in the toxic properties of soil during subsequent remediation stages.

#### 2.3.1. Soil Phytotoxicity Tests Using the Phytotoxkit^TM^ Test

The phytotoxicity tests conducted showed that the sample of the petroleum-contaminated soil (soil G6) before biodegradation was toxic to the tested plants: *Sorghum saccharatum*, *Lepidium sativum* and *Sinapis alba*. Out of this group, the plant most sensitive to pollution (TPH and PAHs) was cress, for which the root growth was inhibited by 43.1% after 72 h of the test. A slightly higher resistance to pollution was recorded for sorghum (40.2%) and mustard (39.7%). The second parameter tested, i.e., the percentage of germinated seeds, ranged from 60% to 90%.

At the end of biodegradation tests performed with the prism method using bioaugmentation with biopreparations B1 and B2, the content of petroleum hydrocarbons in the soil decreased, reducing its toxicity, as confirmed by the results of the second phytotoxicity test. For soil G6-3B1, the average increase in root length ranged from 21.5 to 35.7 mm for *Sorghum saccharatum*, from 30.2 to 47.2 mm for *Lepidium sativum* and from 40.2 to 59.8 mm for *Sinapis alba*. In the case of soil inoculated with biopreparation B2, the average root length was 39.9 mm for *Sorghum saccharatum*, 52.4 mm for *Lepidium sativum* and 66.3 mm for *Sinapis alba*. The inhibition of root growth ranged from 25.25% to 31.59% for soil G6-3B1 and from 16.88% to 24.06% for soil G6-3B2. Additionally, in both soils, *Sorghum saccharatum* and *Sinapis alba* germinated 100%, whereas the percentage of germination of *Lepidium sativum* was 70% in soil inoculated with biopreparation B1 and 80% in soil inoculated with biopreparation B2.

**Figure 11:**
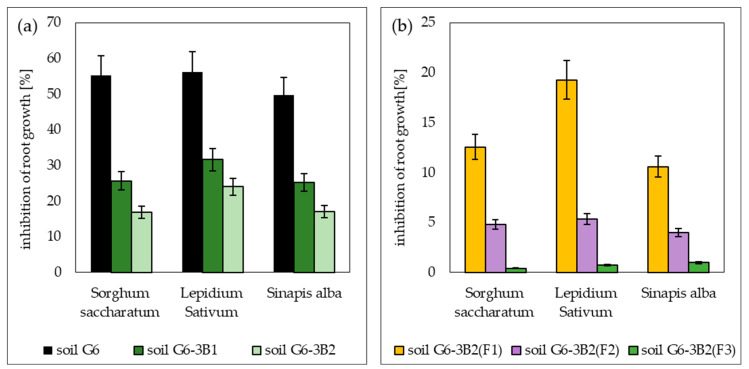
Results of the Phytotoxkit^TM^ test (**a**) carried out for the original soil and after biodegradation tests with the use of biopreparations B1 and B2, (**b**) carried out for soil after completion of phytoremediation tests under the three tested systems (repetition number *n* = 7–10, *p* < 0.05).

As a result of phytoremediation, the best reduction in toxic soil properties was achieved in the system inoculated with biopreparation B2 with added γ-PGA, where the average root length was 47.8 mm for *Sorghum saccharatum*, 68.5 mm for *Lepidium sativum* and 79.2 mm for *Sinapis alba*. Root growth inhibition ranged from 0.42% to 1%. Slightly worse test results were achieved in soil G6-3B2(F2), where the average root length of the tested plants ranged from 45.7 mm (*Sorghum saccharatum*) to 76.8 mm (*Sinapis alba*), and root growth inhibition ranged from 4.00% to 5.36%. The effect of soil remediation by phytoremediation was worst by a wide margin in system 1, where the average root length of *Sorghum saccharatum*, *Lepidium sativum* and *Sinapis alba* was 41.2 mm, 55.7 mm and 69.45 mm, respectively, and the average growth inhibition ranged from 10.6 to 19.28. A summary of the Phytotoxkit^TM^ test results conducted during the remediation of G6 soil is included in Figure 11.

#### 2.3.2. Soil Toxicity Tests Using the Ostracodtoxikit^FTM^ Test

The Ostracodtoxikit^FTM^ tests showed that the initial soil G6 had toxic properties, as evidenced by the increase in the mortality of Heterocypris incongruens crustaceans to 46.67% relative to the control soil (clean soil) and inhibition of the growth of ostracods by 37.1%. The bioremediation of soil by inoculation with biopreparations made with native microorganisms reduced its toxicity to ostracods. In soil G6-3B1, the mortality of the test organisms was recorded at 25.00%, and the average growth inhibition decreased to 16.3%, while in soil G6-3B2, the mortality of ostracods decreased to 18.33%, and the average growth inhibition decreased to 11.7%.

**Figure 12:**
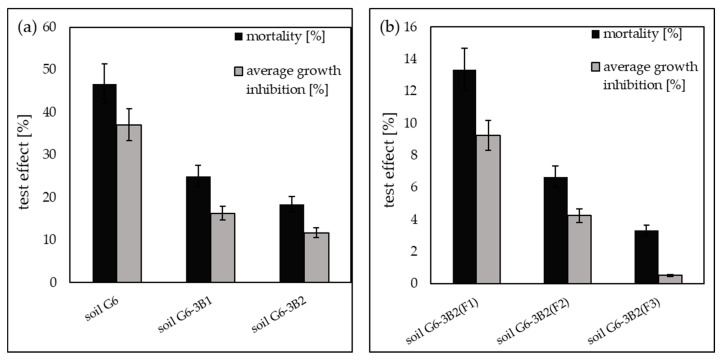
Results of the Ostracodtoxkit^FTM^ test (**a**) carried out for the original soil and after biodegradation tests with the use of biopreparations B1 and B2, (**b**) carried out for soil after completion of phytoremediation tests under the three tested systems (repetition number *n* = 7–10, *p* < 0.05).

Soil toxicity tests using the Ostracodtoxikit^FTM^ test conducted after the completion of phytoremediation of the soil using *Zea mays* showed the highest decrease in the average mortality of *Heterocypris incongruens* and average growth inhibition in soil G6-3B2(F3) of 3.33% and 0.52%, respectively. Slightly higher ostracod mortality and growth inhibition were recorded in soil G6-3B2(F2), namely 6.67% and 4.25%, respectively, while the parameters tested in soil G6-3B2(F1) were 13.33% (mortality) and 9.24% (average growth inhibition). A summary of the results of the Ostracodtoxkit^FTM^ test conducted during the remediation of G6 soil is included in Figure 12.

#### 2.3.3. Soil Toxicity Tests Using the Microtox^®^SPT Test

**Figure 13:**
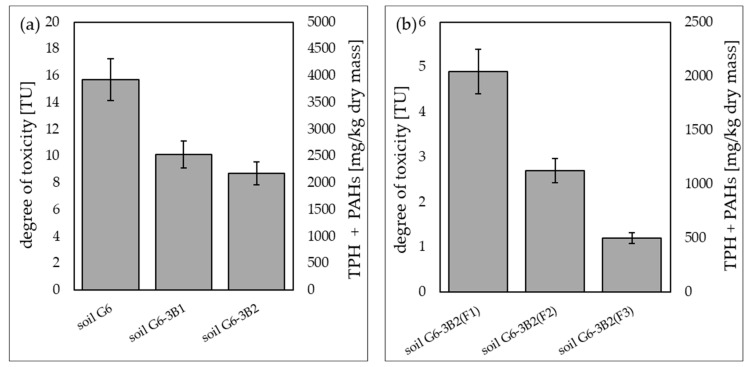
Results of the Microtox^®^SPT test (**a**) carried out for the original soil and after biodegradation tests with the use of biopreparations B1 and B2, (**b**) carried out for soil after completion of phytoremediation tests under the three tested systems (repetition number *n* = 7–10, *p* < 0.05).

Soil toxicity tests on the trophic level of decomposers were conducted using the Microtox^®^SPT test (for solid phase), enabling direct contact of luminescent bacteria (*Vibrio fischeri*) with the tested soil sample. The tests showed that soil contaminated with petroleum products (TPH, PAHs) before the biodegradation process was highly toxic, as indicated by the toxic units TU = 15.7. As a result of inoculation with biopreparation B1, TU was reduced in the Microtox^®^SPT test to 10.1, while inoculation with biopreparation B2 reduced TU to 8.7. The phytoremediation treatments performed under the tested systems additionally reduced TU to 4.9 in soil G6-3B2(F1), 2.7 in soil G6-3B2(F2) and 1.2 in soil G6-3B2(F3). A summary of the results of the Microtox^®^SPT test conducted during the remediation of G6 soil is included in Figure 13.

## 3. Discussion

The bioremediation tests using biopreparations made with native microorganisms showed differences in the efficiency of biodegradation of both aliphatic hydrocarbons and polycyclic aromatic hydrocarbons, depending on the composition of the applied biopreparation. The use of biopreparation B2 enhanced with selected species of fungi resulted in a greater reduction in aliphatic hydrocarbons in the soil than the use of B1 bacterial biopreparation [69,70]. The presence of fungi in the biopreparation had the biggest impact on the efficiency of biodegradation of aliphatic hydrocarbons with a carbon chain length of n-C_22_–n-C_30_ and heavy hydrocarbons with a carbon chain length of n-C_31_–n-C_36_ [27,55]. This is confirmed by studies by other researchers, indicating that *Penicillium* sp., *Candida* sp., *Aspergillus* sp. are good degraders of aliphatic hydrocarbons with carbon chain lengths of C_10_–C_36_ [27,28,29,30,31]. The available research indicates that most microorganisms can metabolise hydrocarbons to a limited extent. Consequently, in order to increase the efficiency of bioremediation of soil contaminated with various groups of hydrocarbons (aliphatic hydrocarbons, PAH), multi-component mixtures with strains of bacteria and fungi with a broad spectrum of action should be used. Additionally, the efficiency of biodegradation of aliphatic hydrocarbons depends on the size and structure of the molecule [55,120]. Aliphatic hydrocarbons with a carbon chain length of n-C_10_–n-C_21_ were the most readily biodegradable as a result of inoculation with biopreparations B1 and B2, whereas heavy hydrocarbons were the most difficult to break down. Hydrocarbons with shorter carbon chains are, therefore, more readily used by microorganisms in metabolic processes. It was generally assumed that the rate of removal of selected groups of aliphatic hydrocarbons from soil inoculated with both the bacterial biopreparation and biopreparation made with bacteria and fungi decreased in the following order: nC_10_–nC_21_> nC_6_–nC_9_ > nC_22_–nC_30_ > nC_31_–nC_36_ [120,121,122,123]. The situation was the same with polycyclic aromatic hydrocarbons. The inoculation of the soil tested with biopreparation B2 produced a higher decrease in PAH concentration after 3 months of tests than inoculation with biopreparation B1. Considering the structure of the hydrocarbon molecule, it is assumed that biodegradation efficiency decreases along with the increase in the number of aromatic rings in the PAH molecule. The rate of removal of polycyclic aromatic hydrocarbons from soil inoculated with biopreparations B1 and B2 decreased in the following order: 2-ring-PAH > 3-ring-PAH > 4-ring-PAH > 5-ring-PAH > 6-ring-PAH [55]. The sequence of removal of individual groups of hydrocarbons from the soil matrix was consistent with the results obtained in previous studies and with the results obtained by other researchers [55,120,121,122,123].

The phytoremediation tests conducted using *Zea mays* demonstrated that the use of plants for the remediation of petroleum-contaminated land enabled a significant decrease in TPH and PAH concentrations in the tested soil. *Zea mays* is tolerant to high concentrations of xenobiotics and has a high rate of pollution accumulation, grows quickly, has large biomass production and is resistant to diseases and pests [124]. Beneficial results were also obtained during the use of maize in phytoremediation tests of heavy metals and petroleum hydrocarbons [97,98,99,124,125,126,127,128]. Most researchers conducting research using Zea mays in bioremediation treatments focus only on the phytoremediation process, not taking into account combined techniques, e.g., phytoremediation supported by inoculation [98,99]. Since the tests were conducted under three systems (system 1—phytoremediation; system 2—phytoremediation enhanced with inoculation with biopreparation B2; and system 3—phytoremediation enhanced with inoculation with biopreparation with added γ-PGA), it was possible to determine the optimum conditions for the remediation of soil from waste pit areas and take into account the impact of bioaugmentation on the effectiveness of the phytoremediation process. The least optimal method was the use of phytoremediation in isolation using *Zea mays*. After 6 months of tests, the efficiency of TPH biodegradation was 22.81%, and the efficiency of PAH biodegradation was 14.48%. The combination of phytoremediation with inoculation with biopreparation B2 enabled a significant increase in the efficiency of biodegradation of TPH and PAHs. The degree of reduction in TPH and PAHs in system 2 was higher than in system 1 by 26.99% and 22.26%, respectively. The most optimal solution was the use of phytoremediation using *Zea mays* in combination with inoculation with biopreparation B2 with added γ-PGA. The use of the γ-PGA additive with biopreparation B2 enabled an additional increase in the efficiency of soil remediation of 16.27% (TPH) and 20.06% (PAHs) relative to system 2. Additionally, the best plant growth was observed in system 3, as indicated by the best development of the root, stem and leaves. The results obtained from chromatographic analyses after the biodegradation process are consistent with the current knowledge, and the use of different systems of phytoremediation tests using *Zea mays* enabled an improvement of the existing methods for remediation of petroleum-contaminated soil using plant organisms [97]. Based on the chromatographic analyses conducted and observations of the morphology of the plants, it was found that the use of microorganisms and additives such as γ-PGA had a significant impact on the efficiency of soil remediation. The microorganisms present in the biopreparation can increase the bioavailability for the pollutant and the conversion of pollutants into forms more readily absorbed by the plants; they can also reduce the concentration of the pollutants to a level tolerated by the plants, as well as the presence of symbiotic interactions between the plant and the bacteria or fungi in the biopreparation [88,90,94,95,96,97]. Additionally, the better growth of plants in systems 2 and 3 may be indicative of the impact of biopreparation on the epuvalisation process, which consists of the plant using the products of bacterial breakdown to increase biomass production [129]. It should also be noted that the efficiency of phytoremediation was significantly affected by the presence of γ-PGA in the biopreparation. The available research indicates that due to its specific structure, γ-PGA is resistant to the action of microorganisms and has strong hygroscopic properties, which enables the maintenance of appropriate soil moisture—e.g., during a drought—has properties, which bind harmful substances, and can be used as an additional source of carbon [110,111,112,113,114].

The efficiency of the applied bioremediation treatments was complemented by toxicological monitoring intended to determine the impact of petroleum pollutants (TPH, PAHs) and indirect metabolites produced during remediation treatments on soil biocenosis based on the toxicological tests conducted. Soil inoculated with biopreparation B2 was less toxic to the indicator organisms after the tests in comparison with soil inoculated with biopreparation B1. The toxicological tests conducted on soil subjected to phytoremediation using *Zea mays* under three tested systems showed the highest decrease in soil toxicity relative to the indicator organisms tested under the system inoculated with biopreparation B2 with added γ-PGA (system 3). The smallest decrease in toxicity in soil subjected to phytoremediation was recorded for system 1. The toxicological tests conducted confirmed the correlation between the efficiency of the removal of petroleum pollutants from the soil matrix and the decrease in its toxicity.

## 4. Materials and Methods

### 4.1. Materials

**Figure 14:**
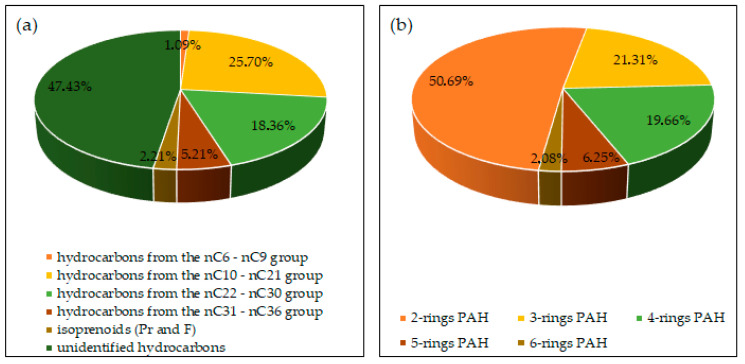
Content (%) of individual components of petroleum pollutants in the soil: (**a**) aliphatic hydrocarbons, (**b**) PAHs (repetition number *n* = 7–10, *p* < 0.05).

The biodegradation tests of petroleum products used soil collected from the G-6 waste pit located in south-eastern Poland (N: 49°40′16.22″, E: 22°04′42.99″). The soil tested had a high concentration of TPH (4527.70 mg/kg ds) and PAHs (10.48 mg/kg ds). The analyses of soil samples for the presence of heavy metals did not indicate any breach of the limits. The content of the individual hydrocarbon groups in the soil tested is shown in Figure 14.

For soil inoculation, two types of biopreparations developed on the basis of autochthonous microorganisms were used in the research. Bacterial biopreparation B1 consisted of the bacterial strains *Dietzia* sp. IN118, *Gordonia* sp. IN101, *Mycolicibacterium frederiksbergense* IN53, *Rhodococcus erythropolis* IN119, *Rhodococcus globerulus* IN113 and *Raoultella* sp. IN109, and biopreparation B2 was enriched with *Aspergillus sydowii*, *Aspergillus versicolor*, *Candida* sp., *Cladosporium halotolerans*, *Penicillium chrysogenum*. The Ambiogel^®^ (Ambioteco Sp. z o.o., Staszów, Poland) containing 5% of pure poly-γ-glutamic acid with varying molecular mass (2000–1000 kDa—30%; 1000–140 kDa—50%; 100 kDa–300 Da—15%; <300 Da—5%) was used as a γ-PGA additive [130]. The phytoremediation tests used seeds of maize (*Zea mays*) (Legutko, Poland) planted in pre-treated soil (after completing biodegradation tests using inoculation with biopreparations) in May 2022.

### 4.2. Methods

#### 4.2.1. Construction of Biopreparations

The bacterial and fungal strains used in this study (bacteria: *Dietzia* sp. IN118, *Gordonia* sp. IN101, *Mycolicibacterium frederiksbergense* IN53, *Rhodococcus erythropolis* IN119, *Rhodococcus globerulus* IN113 and *Raoultella* sp. IN109; fungi: *Aspergillus sydowii*, *Aspergillus versicolor*, *Candida sp*., *Cladosporium halotolerans, Penicillium chrysogenum*) came from the hydrocarbon-degrading microbial collection of the Department of Microbiology (at the Oil and Gas Institute—National Research Institute, Poland). Some of them were previously tested, and their hydrocarbon-degrading capabilities are well established [55,56]. Bacterial strains were phylogenetically identified following 16S rDNA sequencing analysis, as described elsewhere [121]. Strain diagnostic features were determined based on microscopic observations, morphology, growth on the selective agar media and biochemical profile (API tests, bioMerieux). The 16S rDNA sequences of bacterial strains were deposited in the NCBI database, and their accession numbers are shown in Table 1 (together with their capabilities of using selected hydrocarbons as a sole carbon and energy source).

The hydrocarbon-metabolising capabilities of all microbial strains were examined based on growth on the mineral medium supplemented with the compounds tested, as described in our previous work and according to the methods of Wrenn and Venosa [131]. Each bacterial strain was grown individually in a nutrient broth supplemented with sodium acetate (0.2%, *w*/*v*), while each fungal strain was grown in a Czapek–Dox broth (both BD Difco™) and incubated at room temperature with shaking at 150 rpm for 24–72 h to obtain density 10^8^–10^9^ cfu/mL. The biopreparations used in this study were constructed by mixing equal volumes of each strain. Then, the obtained mixed culture was kept in a nutrient broth supplemented with sodium acetate (0.2%, *w*/*v*) and sucrose and occasionally examined for the presence of all introduced strains. Table 2 presents the putative genes involved in petroleum hydrocarbon degradation found in genomes of the closest relatives of all bacterial strains used in this study [55,56].

#### 4.2.2. Identification of Fungal Strains by MALDI-TOF MS

Matrix-assisted laser desorption/ionisation time-of-flight mass spectrometry (MALDI-TOF MS) was used to identify specific fungal strains and was performed at the Jagiellonian Centre of Innovation (Kraków, Poland). A colony from a fresh overnight culture was used for sample preparation to obtain the mass spectrometer measurement. The material was suspended in 300 μL double-distilled water, following which, 900 μL ethanol was added, and the components were mixed well; thereafter, the mixture was centrifuged (2 min, 9000× *g*), and the supernatant was removed. For sample extraction, 40 μL of formic acid (70% in water) was added to the fungal pellet, following which, the components were mixed thoroughly, and 40 μL of acetonitrile was added. After performing centrifugation at 9000× *g* for 2 min, 1 μL of the supernatant containing the fungal extract was transferred to a well of the 96-well MALDI Biotarget plate (Bruker Daltonics, Bremen, Germany) and was allowed to dry at room temperature. Subsequently, the sample was overlaid with 2 μL of the MALDI matrix solution (α-cyano-4-hydroxy-cinnamic acid) and air-dried again. The measurements were performed using the MALDI BioTyper 2.0 Microflex LT system (Bruker), using the manufacturer’s recommended settings. In order to perform microorganisms identification, the raw protein spectral data were imported into the MALDI BioTyper 2.0 software (Bruker) and were analysed via standard pattern matching (with default parameter settings) against the spectral data in the BioTyper database (Bruker). Parameters such as the mass-to-charge ratio and peak intensity were considered to produce a matching score, which was subsequently used to rank the results. In these studies, the identification criteria were as follows: a score of 2.000 and above was considered sufficient for correct species identification; score values of 1.700 to 1.999 confirmed genus identification; and scores lower than 1.700 indicated no identification.

#### 4.2.3. TPH and PAH Analyses

The determination of TPH and PAH was carried out according to the procedures developed by the Oil and Gas Institute—State Research Institute (Kraków, Poland).

The TPH analysis was conducted using the gas chromatography method, employing the Clarus 500 chromatograph (Perkin Elmer, Waltham, MA, USA) with a flame ionisation detector (FID). Before the chromatographic analysis could be conducted, it was necessary to correctly prepare the soil samples, including the following:

- drying soil samples to an air-dry condition;
- isolating the analytes using ultrasound-assisted extraction with dichloromethane (Chempur, Piekary Śląskie, Poland);
- purifying the extract using the dSPE method, employing Florisil columns Chromabond No. 30081 (Macherey-Nagel, Düren, Germany);
- concentrating the extract to a volume of 1 mL in a rotary evaporator (Chemland, Stargard, Poland).

**Figure 15:**
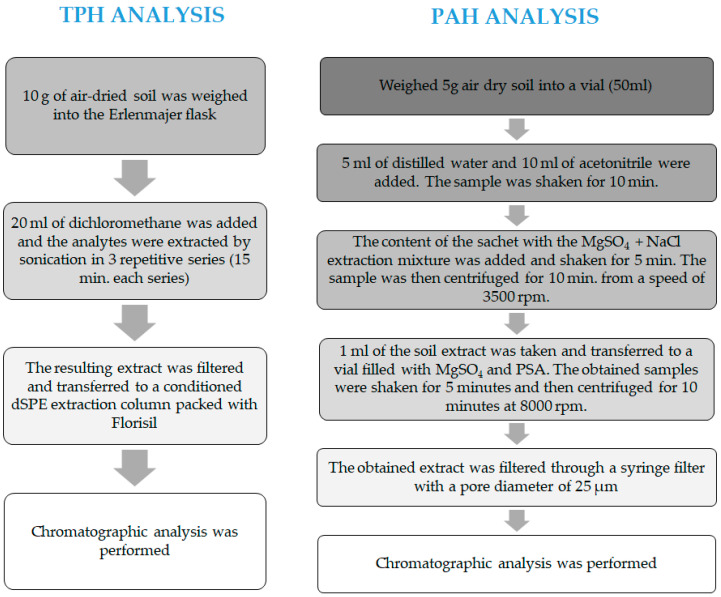
Scheme for determination of TPH and PAHs in soil samples.

Then, the produced extract was subjected to a chromatographic analysis using the following operating parameters of the chromatograph: capillary column of fused silica (RTX-1:30 m × 0.53 mm) (Restek, Bellefonte, PA, USA); using the following temperature parameters: injector temperatures = 290 °C, FID temperature = 320 °C; using the following oven temperature programme: 30 °C—isothermal run for 2 min, 30 °C to 105 °C—temperature increase at a rate of 10 °C/min, 105 °C to 285 °C—temperature increase at a rate of 5 °C/min, 285 °C—isothermal run for 5 min [55,56]. The diagram of the TPH analysis is shown in Figure 15.

The PAH analysis was conducted using the HPLC liquid chromatography method, employing the Vanquish Core chromatography set (Thermo Scientific, Waltham, MA, USA). The analysis of the quantitative and qualitative determination of 16 PAHs in the soil samples required the preparation of an analytical sample, which included the following:

- drying soil samples to an air-dry condition;
- isolating the analytes (PAHs) using the QuEChERS method;
- purifying the extract using the dSPE method with MgSO_4_ and PSA vials No. JO3937 (Interchim, Montluçon Cedex, France).

Then, the produced extract was subjected to a chromatographic analysis with the following operating parameters of the equipment: NUCLEODUR C_18_ PAH column, 125 × 4 mm, 3 μm (Marcherey-Nagel, Düren, Germany); detector: UV-VIS; fluorometric (FLD) dispenser: automatic; dispensing volume: 10 μL; eluents: A-methanol (POCH S.A., Poland) 70%, B-acetonitrile (POCH S.A., Gliwice, Poland); flow rate: 1.5 mL/min; gradient: 20% B for 1.5 min, 20% to 50% B for 1.5 min, 50% to 100% B for 1 min, 100% B for 1 min, 100% to 0% B for 3 min, 100% A for 3 min [55]. The diagram of the PAH analysis is shown in Figure 15.

The specific method for the determination of TPH and PAHs in soil samples was described elsewhere [55,56].

#### 4.2.4. Ecotoxicological Analyses

Soil toxicology analysis was performed using commercially available toxicology tests Phytotoxkit ^TM^ (MicroBioTests Inc., Nazareth, Belgium), Ostracodtoxkit ^TM^ (MicroBioTests Inc., Nazareth, Belgium), Microtox^®^ Solid Phase Test (SDI, Newark, DE, USA). The use of living organisms as bioindicators belonging to various taxonomic groups (higher plants, crustaceans, bacteria) representing all trophic groups (producers, consumers and decomposers) allowed for a comprehensive assessment of the effectiveness of the process for cleaning soil contaminated with oil derivatives.

Toxicological tests were carried out in soil samples taken before process purification (initial soil—G6), after completion of biodegradation with biopreparations B1 (soil G6-3B1) and B2 (soil G6-3B2) and after completion of phytoremediation under the systems tested (soil G6-3B2(F1), G6-3B2(F2), G6-3B2(F3)). A detailed discussion of the toxicological tests used and the description of the procedures for performing them are provided in Supplementary Materials and elsewhere [55,56].

#### 4.2.5. Data Analysis and Statistical Information

Statistical analysis was performed with Statistica 14.0. Standard deviation (SD), standard deviation (RSD) (%) and Pearson correlation coefficient were calculated. The obtained data were first tested for normal distribution. Then, they were analysed with one-way ANOVA, followed by a post hoc pairwise Tukey test (when the ANOVA produced significant results). Significance was set at *p* < 0.05 [55,56].

### 4.3. Experiment Description

#### 4.3.1. Biodegradation by Inoculation

Biodegradation tests using inoculation with biopreparations made with native microorganisms were conducted using the ex situ prism method. The layer of gravel used for the placement of perforated pipes responsible for the supply of air to the site was covered with soil collected from the G6 mining pit in the form of two identical prisms with a weight of 60 kg each. The soil was tested for the presence of petroleum products, the concentration of heavy metals and the C:N:P ratio. To ensure the optimum conditions for the development of microorganisms in the biopreparation, the C:N:P ratio was adjusted via supplementation with mineral fertiliser Azofoska (Inco, Warszawa, Poland), and the pH of the soil was adjusted using fertiliser lime (Biovita, Tenczynek, Poland). The soil prepared in this way had a C:N:P ratio of 100:10:1 and a pH of 7.5. Then, each prism was inoculated with biopreparation B1 (prism No. 1) or biopreparation B2 (prism No. 2) in the amount of 6 L per prism. Biodegradation tests using inoculation with biopreparations made with native microorganisms were conducted for 3 months at a constant temperature of 25 °C and constant humidity (20% to 25%) [55,56]. A diagram of the stand for testing the process of biodegradation is shown in Supplementary Materials (Figure S1).

#### 4.3.2. Phytoremediation

The phytoremediation tests were conducted using the pot method on a semi-technical scale. Pots with a volume of 10 L were filled with soil (15 kg), which had been pre-treated by inoculation with the biopreparation selected based on biodegradation tests of petroleum pollutants (biopreparation B2). The soil tested had a known concentration of petroleum pollutants (TPH = 2640.83 mg/kg ds and PAHs = 6.84 mg/kg ds), a suitable C:N:P ratio of 100:10:1 and humidity of 20%, which enabled optimum conditions for the remediation process. Then, 8 seeds of maize were sown in each pot, and, depending on the system tested, 1 litre of water, biopreparation B2 or biopreparation B2 with added γ-PGA was added to each pot.

- system 1—soil G6-3B2(F1)—(soil G6-3B2 + *Zea mays*);
- system 2—soil G6-3B2(F2)—(soil G6-3B2 + biopreparation B2 + *Zea mays*);
- system 3—soil G6-3B2(F3)—(soil G6-3B2 + biopreparation B2 with added γ-PGA + *Zea mays*).

Each of the systems tested was covered with foil until the plants germinated. The phytoremediation tests were carried out for 6 months in the actual conditions to test the possibility of using the proposed method in field conditions (waste pits). The layout of the site for phytoremediation tests is shown in Supplementary Materials (Figure S2).

## 5. Conclusions

Petroleum hydrocarbons are the leading group of pollutants generated by the oil and gas extraction industry. Due to their carcinogenic, neurotoxic and mutagenic properties, oil-derived pollutants pose a serious threat to the environment, as well as to the health and life of humans and animals. For this reason, it is extremely important to develop effective methods of cleaning up oil-contaminated areas. In our work, we indicated the enormous potential for bioremediation of soils contaminated with TPH and PAHs using bioaugmentation and bioaugmentation-assisted phytoremediation techniques. Biodegradation studies conducted using the ex situ piling method with the use of two types of biopreparations showed that the addition of fungi to the biopreparation significantly increases the biodegradation efficiency of both aliphatic and aromatic hydrocarbons. This confirms the assumption that the use of more strains of microorganisms in the biopreparation provides it with a broad spectrum of activity. Currently, more and more research is being conducted on accelerating the process of bioremediation of soils contaminated with toxic substances by combining several remediation techniques or their modifications. The combination of phytoremediation and bioaugmentation enabled an additional reduction in the concentration of petroleum hydrocarbons in the soil tested and an increase in the efficiency of its treatment of 49.08% (TPH) and 40.74% (PAH). The use of γ-PGA as an additive to the biopreparation allowed for increased efficiency of phytoremediation supported by bioaugmentation of 16.27% (TPH) and 20.06% (PAH). The assessment of the effectiveness of the applied bioremediation treatments included the results of toxicological tests carried out using the Phytotoxkit ^TM^, Ostracodtoxkit ^TM^, Microtox^®^ Solid Phase test kits. Depending on the test used, soil toxicity varied, but it was correlated with the concentration of pollutants contained in the soil.

Promising results obtained during biodegradation tests with the use of biopreparations B1 and B2 and phytoremediation (using *Zea mays* as a phytoremediant) supported by bioaugmentation may in the future be a step forward in the methods of bioremediation of soils contaminated with TPH and PAHs and may be used in field conditions.

## Figures and Tables

**Table 1:** Hydrocarbon-degrading capabilities of bacterial strains and fungal strains comprising biopreparations B1 and B2.

Strain	NCBI Accession Number	nC_18_H_38_	Izo-C_19_H_40_	Tol + Xyl	N	A	Phen	Fluo	F	Pir
*Dietzia* sp. IN118	KT923327	+	+	+/−	+	−	+	−	−	+
*Gordonia* sp. IN101	KT923337	+	+	+	+	−	+/−	−	−	−
*Mycolicibacterium frederiksbergense* IN53	JN572675	+	+	−	+	+	+	−	−	+
*Raoultella* sp. IN109	KT923339	+	+	+/−	+	−	+	−	−	+/−
*Rhodococcus erythropolis* IN119	KT923331	+	+	+	+	−	−	−	−	−
*Rhodococcus globerulus* IN113	KT923347	+	+	+	+	+/−	+/−	+/−	−	+/−
*Aspergillus* *sydowii*	n/a	+	n/t	−	−	−	+	n/t	n/t	n/t
*Aspergillus versicolor*	n/a	+	n/t	+	+	+	+	n/t	n/t	n/t
*Candida* sp.	n/a	+	n/t	+/−	+	−	−	n/t	n/t	n/t
*Cladosporium halotolerans*	n/a	+	n/t	−	+	+	+	n/t	n/t	n/t
*Penicillium* *chrysogenum*	n/a	+	n/t	+	+	−	−	n/t	n/t	n/t

iso-C_19_H_40_: pristane, Tol: toluene, Xyl: mixture of xylenes, N: naphthalene, A: anthracene, Phen: phenanthrene, Fluo: fluorene, F: fluoranthene, Pir: pyrene, +: growth, −: no growth, +/−: ambiguous observation, n/a: not applicable, n/t: not tested.

**Table 2:** Identification of bacterial strains used in biopreparations B1 and B2.

Strain	The Closest Relative Based on 16S rRNA Accession Number (% of Identity) *	The Closest Relative for Which the Genome Sequence Is Available in NCBI GenBank, Accession Number (% of Identity) *	Putative Gene Encoding for the Enzymes Catalysing the Breakdown of Hydrocarbons
*Dietzia* sp. IN118	*Dietzia* sp. LJ3 MG049763 99.93%	*Dietzia kunjamensis* 313 CP099712 99.78%	Alkane 1-monooxygenase, aromatic ring-hydroxylating dioxygenase subunit alpha
*Gordonia* sp.IN101	*Gordonia* sp. Tm-B24 MT533993 99.63%	*Gordonia terrae* RL-JC02 CP049836 99.63%	Alkane 1-monooxygenase, pentachlorophenol monooxygenase, naphthalene 1,2-dioxygenase subunit alpha (2 copies), 2,3-dihydroxybiphenyl 1,2-dioxygenase (2 copies)
*Mycolicibacterium frederiksbergense* IN53	*Mycolicibacterium* *frederiksbergense* DSM 44346 (typical strain) NR_025393.1 99.58%	*Mycolicibacterium* *frederiksbergense* LB 501T 99.58%	Alkane 1-monooxygenase (2 copies), pentachlorophenol monooxygenase, naphthalene 1,2-dioxygenase subunit alpha (2 copies), 2,3-dihydroxybiphenyl 1,2-dioxygenase (2 copies)
*Rhodococcus erythropolis* IN119	*Rhodococcus erythropolis* KD-1 CP050124 99.42%	*Rhodococcus erythropolis* KD-1 CP050124 99.42%	Alkane 1-monooxygenase (5 copies), pentachlorophenol monooxygenase, cyclohexanone monooxygenase (2 copies), biphenyl 2,3-dioxygenase (2 copies), 2,3-dihydroxybiphenyl 1,2-dioxygenase (2 copies)
*Rhodococcus* *globerulus* IN113	*Rhodococcus globerulus* D757 CP079698 99.86%	*Rhodococcus globerulus* D757 CP079698 99.86%	Alkane 1-monooxygenase (3 copies), 2,3-dihydroxybiphenyl 1,2-dioxygenase (2 copies), aromatic ring-hydroxylating dioxygenase subunit alpha (7 copies)
*Raoultella* sp. IN109	*Raoultella planticola* SCLZS62 CP082168 99.08%	*Raoultella planticola* SCLZS62 CP082168 99.08%	aromatic ring-hydroxylating dioxygenase subunit alpha (2 copies)

* the data presented concern the data gathered in the NCBI GenBank database (June 2023). Therefore, strain IN118 is presented here as *Dietzia* sp. instead of *Dietzia maris* (as it was originally identified).

## Data Availability

Not applicable.

## Supplementary Materials

The following supporting information can be downloaded at: https://www.mdpi.com/article/10.3390/molecules28166104/s1, Table S1—Total content of selected groups of TPH and PAHs in soil G6 and after the biodegradation process with the use of biopreparation B1 (soil G6-3B1) and B2 (soil G6-3B2), Table S2—Total content of selected groups of TPH and PAHs in soil after the phytoremediation process in the tested systems, Ecotoxicological analyses—description of the methodology, Figure S1—Stand for testing the process of biodegradation of petroleum hydrocarbons, Figure S2—Stand for testing the process of phytoremediation of soil G6-3B2.
