# Supplementary material for: Oil-Contaminated Soil Remediation with Biodegradation by Autochthonous Microorganisms and Phytoremediation by Maize (Zea mays)
Source: Molecules. 2023 Aug 17;28(16):6104. doi: 10.3390/molecules28166104 (PMC10459520; doi:10.3390/molecules28166104)
Supplement: Supplementary file 1 [file molecules-28-06104-s001.zip › molecules-2518722-supplementary.pdf]

molecules

Oil-contaminated soil remediation with the biodegradation by autochthonous microorganisms and phytoremediation by maize (*Zea mays*)

Katarzyna Wojtowicz [1]*, Teresa Steliga [1], Piotr Kapusta [1], Joanna Brzeszcz[1]

[1] Oil and Gas Institute – National Research Institute, 31-503 Krakow, ul. Lubicz 25 A, Poland
* Correspondence: wojtowiczk@inig.pl *

Table S1 Total content of selected groups of TPH and PAHs in soil G6 and after the biodegradation process with the use of biopreparation B1 (soil G6-3B1) and B2 (soil G6-3B2) , (repetition number n=7-10, p<0,05)

Hydrocarbons	Content ± SD (mg/kg dry mass soil)		
		After 90 Days	
	Initial Soil G6	Soil G6-3B1<br>soil G6 inoculated with biopreparation B1	Soil G6-3B2<br>soil G6 inoculated with biopreparation B2
TPH	4527,7 ± 316,94	3090,60 ± 216,34	2640,83 ± 184,94
Unidentified aliphatic hydrocarbons	2147,6± 150,33	1472,89 ± 103,10	1332,13 ± 93,33
$\sum nC_6–nC_9$	49,30 ± 4,93	19,79 ± 1,98	13,42 ± 1,34
$\sum nC_{10}–nC_{21}$	1163,60 ± 116,36	616,19 ± 61,62	455,35 ± 45,50
$\sum nC_{22}–nC_{30}$	831,30± 83,13	676,74 ±	557,34±
$\sum nC31–nC_{36}$	235,80± 23,58	211,41 ± 21,14	195,89± 19,59
isoprenoids	100,10 ± 10,01	94,07 ± 9,41	86,70 ± 8,67
∑PAHs	10,48 ± 1,05	7,61 ± 0,76	6,84 ± 0,68
∑two -ring PAHs	5,31 ± 0,53	3,48 ± 0,65	2,99 ± 0,03
∑ three - ring PAHs	2,23 ± 0,22	1,64 ± 0,15	1,50 ± 0,15
∑ four- ring PAHs	2,06 ± 0,21	1,71 ± 0,17	1,62 ± 0,16
∑ five- ring PAHs	0,66 ± 0,07	0,58 ± 0,06	0,55 ± 0,06
∑ six-ring PAHs	0,22 ±0,02	0,20 ± 0,02	0,19 ± 0,02

Table S2 Total content of selected groups of TPH and PAHs in soil after the phytoremediation process in the tested systems, (repetition number n=7-10, p<0,05)

Hydrocarbons	Content ± SD (mg/kg dry mass soil)		
	After 180 Days		
	System 1	System 2	System 3
TPH	2023,58± 141,65	1344,83± 94,14	915,16± 64,06
Unidentified aliphatic hydrocarbons	1043,21± 73,02	686,45± 48,05	523,97 ± 36,68
∑nC_6–nC_9	7,11 ± 0,71	3,48 ± 0,35	1,82 ± 0,18
∑nC_{10}–nC_{21}	284,98 ± 28,50	124,81 ± 12,48	48,59 ± 4,86
∑nC_{22}–nC_{30}	448,64 ± 44,86	310,94 ± 31,07	166,71 ± 16,67
∑nC31–nC_{36}	175,17 ± 17,52	147,96 ± 14,80	107,98 ± 10,80
isoprenoids	79,47 ± 7,95	71,19 ± 7,12	66,11 ± 6,61
∑PAHs	5,58 ± 0,56	4,05 ± 0,41	2,68 ± 0,27
∑two -ring PAHs	2,11 ± 0,21	1,36 ± 0,14	0,63 ± 0,06
∑ three - ring PAHs	1,29 ± 0,13	0,87 ± 0,09	0,55 ± 0,06
∑ four- ring PAHs	1,48 ± 0,15	1,23 ± 0,12	0,99 ± 0,01
∑ five- ring PAHs	0,52 ± 0,05	0,44 ± 0,04	0,38 ± 0,04
∑Six-ring PAHs	0,18 ± 0,02	0,15 ± 0,02	0,13 ± 0,01

Ecotoxicological analyses –description of the methodology

Phytotoxkit™ test (MicroBioTests Inc., Belgium)

Chronic toxicity assessment test *Phytotoxkit™* is based on the evaluation of germination and early growth of plants (root elongation inhibition measurement. Three types of plants selected according to germination rate and root growth rate are used in the test, which allows making a complete determination in 3 days of incubation: monocotyledonous - sorghum *(Sorghum saccharatum),* and dicotyledonous - cress *(Lepidium sativum)* and white mustard *(Sinapis alba).* The determination process was carried out using three repetitions for each test plant. The tests were performed on transparent polystyrene test plates. *Incubation conditions:* temperature T = 25°C in darkness, incubation time t = 72 h. *Test reaction:* inhibition of germination and early root growth.

The inhibition in seed germination and root growth was calculated according to a following equation:

$$I = \frac{(A-B)}{B} \cdot 100\%$$

where: $\boldsymbol{I}$ *– inhibition (%),* $\boldsymbol{A}$ *– seed germination or root length in control soil,* $\boldsymbol{B}$ *– seed germination or root length in test soil*

Ostracodtoxkit(F)™ (MicroBioTest Inc. Belgium)

Ostracodtoxkit(F) test belongs to direct contact tests of chronic toxicity estimation with the use of crustaceans *Heterocypris incongruens*. The direct contact test is done with the

application of young bottom crustaceans (*Heterocypris incongruens*), which hatched from cysts during 52 hours (according to the producer's procedure). The test is carried out on 6-hole polystyrene microplates. A standardised nutrient is an algae suspension, which is applied to the suspension in portions of 2 ml. Portions of 10 ostracods are added to each cell of the microplate. 0.5 g of a control soil is applied to the row A, whereas 0.5 g of the soil sample is added to the consecutive rows. Incubation lasts for 6 days in a temperature of 25°C. Results reading consists of estimation of life microforms amount in each hole of the microplate and measurement of their length. Growth inhibition of *H. incongruens* was calculated as

$$GI = 100 - \left(\frac{A}{B} \cdot 100\%\right)$$

where: A – ostracods length in tested soil, B – ostracods length in reference soil

Microtox® Solid Phase Test (SDI, USA)

Microtox Test created in the USA in 1979 as the first bioindication test, combines typical bioindication and analytic precision. Luminescence bacteria *Vibro fisch*eri, which uses about 10% of its metabolism in order to emit light, was used as a bioindicator. In the presence of toxical substances, there is decrease in luminescence and increase in general toxicity of a sample. In an electron transportation system of the bacteria, lucipherasa enzyme (alcane oxygenaze) catalyses oxidation of a reduced substrate (reduced flavine mononucleotide, riboflavin phosphate or flavine adenin dinucleotide) and during this process luminescence, which can be measured with a photometer, takes place. The obtained substrates of this reaction are oxygen and long-chain aldehyde. In the presence of substances that have negative influence on cell metabolism, the decrease in luminescence of bacteria is immediate.

Test Microtox®, produced by SDI Company (USA), enables a direct contact of luminescence bacteria *Vibro fischeri* with a wastewater sample, which leads not only to determination of substances dissolved in water, but also to recognition of lipophilicity systems and poorly dissolved systems in water. Lyophilised bacteria *Vibro fischeri* can be stored in a period of a year in temperature of -20°C and then used for testing immediately after suspending in deionised water. A tests with serial dilutions of the wastewater were done according to a standard procedure and results were obtain in Delta Tox analyser. In this analysis, the concentration of wastewater, which reduces the luminescence by 50%, was determined (half maximal effective concentration, EC_{50}). In this presentation a lower of EC_{50} denotes a higher toxicity. To simplify this interpretation, $EC_{50'}$ values were converted to Toxicity Units (TU) in which a higher value denotes a higher toxicity according to the following formula.

$$TU = \frac{1}{EC_{50}} \cdot 100$$

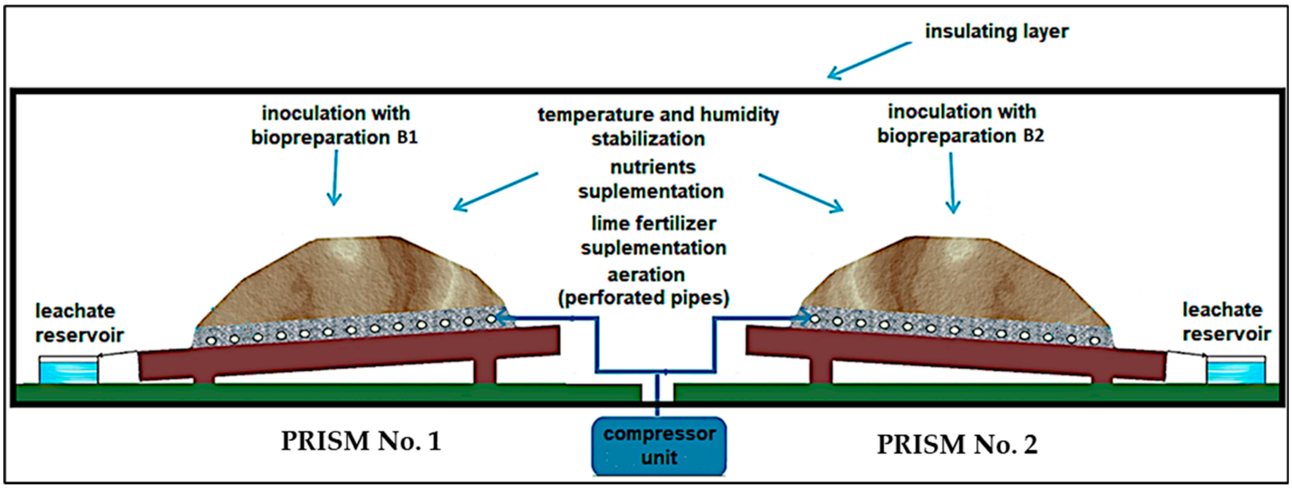

Figure S1. Stand for testing the process of biodegradation of petroleum hydrocarbons.

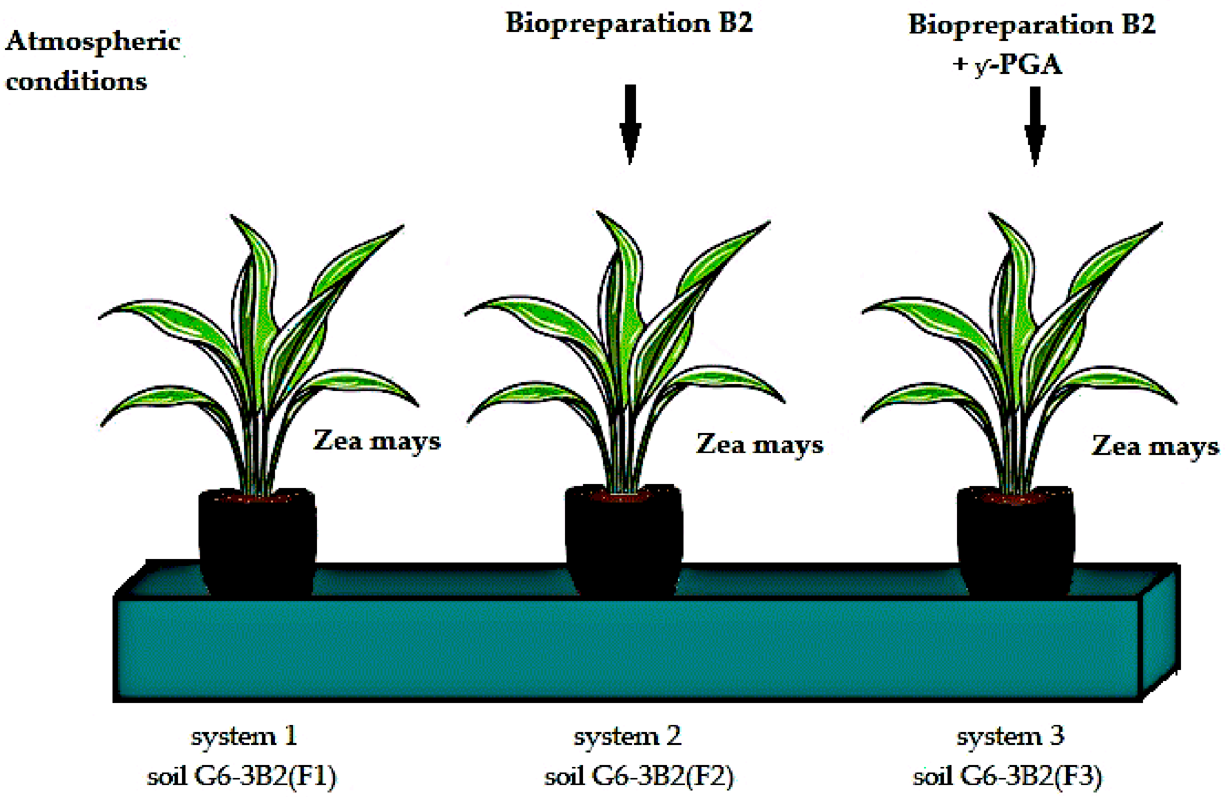

Figure S2. Stand for testing the process of phytoremediation of soil G6-3B2
